# National surveys of patient experiences with addiction services in Norway: do employees use the results in quality initiatives and are results improving over time?

**DOI:** 10.3389/frhs.2024.1356342

**Published:** 2024-11-12

**Authors:** Mona Haugum, Hilde Hestad Iversen, Oyvind Bjertnaes

**Affiliations:** ^1^ Department for Health Services Research, Division for Health Services, The Norwegian Institute of Public Health, Oslo, Norway; ^2^ Faculty of Medicine, Institute of Health and Society, University of Oslo, Oslo, Norway

**Keywords:** patient satisfaction, patient experience, quality of health care, employees’ viewpoints, quality improvement, health services research, survey

## Abstract

**Introduction:**

The experiences of patients receiving health care constitute an important aspect of health-care quality assessments. One of the purposes of the national program of patient-experience surveys in Norway is to support institutional and departmental improvements to the quality of local health-care services. This program includes national surveys of patients receiving interdisciplinary treatment for substance dependence performed four times between 2013 and 2017. The aims of this study were twofold: (i) to determine the attitudes of employees towards these surveys and their use of the survey results, and (ii) to identify changes in patient experiences at the national level from 2013 to 2017.

**Material and methods:**

Employees were surveyed one week prior to conducting cross-sectional patient experience surveys. One-way ANOVA and chi-square tests were used to assess differences between years, and content analysis was applied to the open-ended comments.

**Results:**

Around 400 employees were recruited in each of the four survey years, and the response rate varied from 61% to 79%. The employees generally reported a positive attitude towards patient-experience surveys, and 40%–50% of them had implemented quality initiatives based on the results of the patient surveys. The mean score for the question on usefulness was higher than 3 (on a Likert scale from 1 to 5 points) for all four surveys. Many employees provided details about the changes that had been made in open-ended comments. The results from the patient-experience surveys demonstrated positive changes over time.

**Discussion:**

The employees had positive viewpoints towards patient-experience surveys, and around half of them had implemented quality initiatives. This implies that employees find such surveys important, and that patient-experience surveys are regarded as useful and actionable. The surveys of patients showed positive changes in their experiences over time. The most-common target areas reported by employees showed clear improvements in patient experiences at the national level.

## 1 Introduction

Measuring and reporting patient experiences and satisfaction have become important components of health-care quality assessments, due to their links with patient safety and clinical effectiveness, and also the desire to provide patient-centred care (1, 2). Such measurements are carried out routinely in health-care systems, including in Norway (3–7). In Norway, secondary health-care organizations, such as hospitals and specialized treatment facilities, including those providing interdisciplinary care for substance dependence, are required to measure patient experiences as part of quality and performance reporting, with the results affecting some of the funding that they receive from the government through a scheme where a share of the budget for the health regions is made dependent on achievement of targets on selected indicators (8).

Many methods can be used to gather such data, with surveys being very common (7, 9). The Norwegian Institute of Public Health (NIPH) conducts research on patient-reported experiences and outcomes, aiming for a more patient-centred health system through surveys and continuous measurements across various patient populations. Several different populations have been surveyed using tested and validated questionnaires, such as the PEQ-ITSD for inpatients receiving interdisciplinary treatment for substance dependence and the PIPEQ-OS in mental-health care (10, 11). Results from previous surveys, including the current, are publicly available for all relevant levels of the health-care system, from the smallest units or departments, throughout the health-care hierarchy to the regional and national level, as basis for use in local quality improvement, hospital management, free patient choice and public accountability.

Even though large-scale patient-experience surveys are conducted frequently worldwide, information about the use of the obtained data in improving the quality of local services is scarce (7, 12). Such surveys are often conducted by an external surveyor who reports back to the health-care institutions being surveyed (7, 12), with little support provided on how to use the data (7). Moreover, little is still known about whether or how the results lead to quality improvements (9). A survey found that half of the employee respondents in Norwegian paediatric departments reported that they had implemented improvements to address the problems identified in a national survey of parents, and that such surveys can be actively utilized in quality-improvement interventions (13).

The Norwegian national patient-experience program includes surveys of patients receiving interdisciplinary treatment for substance dependence, which have been conducted four times during 2013–2017, with the first three being annual measurements, and with a small delay for the last, being conducted in 2017 (14–17). The questionnaire used in the patient surveys has been developed and tested using standardized methods to ensure its validity and reliability (10). The surveys have been mandatory for all public residential institutions as well as private residential institutions that have contracts with the regional health authorities, institution here being defined as the most granulated level of health care, and often underlying a department, public hospital, or private organization, constituting the levels of health-care system over institution in the field of interdisciplinary treatment for substance dependence. All patients aged 16 years or older, who were receiving residential treatment for substance dependence were invited to participate in the surveys. Detoxification units and patients treated for gambling addiction were excluded. In addition, patients could be excluded based on ethical considerations by personnel at each institution. Unlike most national surveys in Norway, the data have been collected while the patients are at the institutions, hence requiring close collaboration between the NIPH and institution employees. Reports were distributed a few months after data collection to all levels of health care following each of the four national surveys. The results from the patient-experience surveys were presented on the particular level of health care service, such as department, institution, hospital trust, regional hospital trust as well as national, given sufficient patient responses (*n* ≥ 5). The reports included descriptives on items and scales, as well as a short introduction with description of the methods and appendices where the scores from each hospital trust were compared with the national mean on the scale scores. The reports were distributed via e-mail from the NIPH to regional health authorities who then forwarded to leaders on the health care levels below. The reports were also published on the NIPH's website. Improvements in patient experiences could be expected if the providers use this information to improve the quality of health care (18).

Conducting a patient-experience survey will not in itself improve the quality of care (19). To facilitate the use of patient-experience data to improve the quality of health care, data need to be reliable, valid and usable (6). This includes favorable attitudes toward such data, as well as an absence of barriers that can hinder quality work that is desirable or needed. Alongside the national patient-experience surveys, we surveyed employees at the institutions about their attitudes towards the surveys and their use of the results in quality improvement. The current study had the following aims: (i) to determine the attitudes of employees towards the national patient-experience surveys and their use of the results when providing interdisciplinary treatment for substance dependence, and (ii) to identify changes in patient experiences at the national level from 2013 to 2017 While individual patient care is crucial, this study focuses on how patient-reported experience measures can be utilized to drive improvements at the institutional and departmental level. To our knowledge, this is the first study involving repeated measurements at the national level including both the experiences of patients with health-care services and the assessment of employees and how they use these experiences to improve the health care quality.

## 2 Material and methods

### 2.1 Data collection

When preparing for data collection in the patient-experience surveys, we first established contact with all public residential institutions as well as private residential institutions that had contracts with the regional health authorities. Each institution named a local project manager that would be the NIPH contact responsible for the data collection at their institution. The patient-experience data were collected using a cross-sectional design on a single day decided by the institutions during a single designated week decided by the NIPH. All patients were asked to complete the surveys on the same day to avoid discussions between patients and potential coordination of responses. The health personnel at each institution were responsible for distributing and collecting the completed questionnaires, making this an on-site survey. The questionnaires were provided to the institutions in pre-packed envelopes, each containing an information sheet, the questionnaire itself, and a return envelope. On the designated day of data collection, the staff were instructed to give one envelope to each patient who consented to participate. Patients were informed that participation was voluntary and that their responses would remain confidential. Returning a completed questionnaire was treated as implied consent to participate, following established procedures. The surveys were conducted anonymously as part of a quality assurance project, and no additional demographic data were collected beyond what was included in the questionnaire. Institutions provided NIPH with the number of eligible patients and reasons for ineligibility, but no further information about the individual respondents (10, 20).

When preparing the data collection amongst employees, we asked every local project manager to recruit the following employees from their institutions to participate in an employee survey: department manager, institution manager, quality advisor, and one or two employees who were responsible for quality assurance and improvement. This would ensure up to five recruited employees from every institution in Norway. To ensure that the local project managers were able to recruit the necessary employees, the NIPH provided them with an electronic information sheet about the survey, as well as instructions to forward an email with this information sheet attached from the NIPH. This approach aimed to facilitate recruitment by establishing the NIPH's authority over the survey and to minimize hierarchical differences within the institution. The recruited employees were provided with a link via email to the employee survey during the week prior to each of the four patient-experience surveys. Non-respondents received up to two reminders during that week.

### 2.2 Questionnaires

The reliability and validity of the questionnaire used in the patient-experience surveys have been reported previously (10). Only small changes have been made in it over the years, with some items added but none removed. The questionnaire used in 2017 comprised 58 closed-ended questions, with response scales mostly ranging from 1 (“not at all”) to 5 (“to a very large extent”). The questionnaire also included two open-ended questions where patients could report the help they received from the current institution and the municipality. Tests of these surveys have yielded 3 quality indicators: (i) treatment and personnel (12 items), (ii) milieu (5 items), and (iii) outcome (5 items) (20).

The employee questionnaire was based on previous studies (13, 21), but modified for use in the present population. The original questionnaire was developed through semi-structured face-to-face interviews with staff at a French hospital that had been conducting patient-experience surveys systematically for several years (21). Content analysis was applied to the results from the interviews to identify important themes to be included in an employee-questionnaire. The original questionnaire comprised 17 closed-ended and 4 open-ended questions. Factor analysis revealed two factors with a Cronbach's α > 0.70 (21). The original version was modified for use in specialized paediatric departments in Norway. The modifications were mostly to ensure that the wording was relevant and understandable in the new setting. The Norwegian questionnaire comprised 18 closed-ended and 6 open-ended questions (13). Some further modifications were necessary to ensure relevance in the current population, i.e., employees at residential institutions. The 2013 questionnaire comprised seven closed-ended questions on attitudes towards patient involvement and patient-experience surveys. It was also added a question on whether their institution had carried out any local patient-experience surveys. Six background questions were also included, i.e., gender, age, how many years they had been employed at the current institution, work description, whether they had a managerial position or not, and professional background. The questionnaires used in the three following employee surveys was expanded into 27 items, adding items on the use and usefulness of the national patient-experience surveys when planning and conducting quality-improvement work. Many of the questions were scored on a 5-point response scale ranging from 1 (“not at all”) to 5 (“to a very large extent”), and most of the remaining questions were scored on a categorical response scale. An additional five open-ended questions allowed the employees to provide information on (i) behavioural changes among the staff after patient surveys, (ii) initiatives taken to improve patient experiences and (iii) barriers for using the results from the patient survey previous year, (iv) comments regarding how the results were presented, and (v) report on whatever topics they felt the questionnaire did not cover.

### 2.3 Analysis

Descriptive statistics are presented for the answers of the patients and employees to the closed-ended questions. Mean and SD values are reported for questions scored on the 5-point response scale. One-way ANOVA with Bonferroni post-hoc correction was used to test differences in continuous variables between years, while chi-square tests were applied to categorical responses. Five questions measuring attitudes towards patient-experience surveys were included in an exploratory factor analysis with principal-axis factoring to ensure a unidimensional scale, and Cronbach's alpha was checked for internal consistency. The scale scores were linearly converted from the questionnaire to a scale ranging from 0 to 100, with a higher score indicating a better outcome. Statistical analyses were performed using SPSS version 25.0.

Content analysis was applied to the open-ended comments in the employee survey. This method was chosen to identify key topics and themes that were described by the employees, and hereby ensure that these were the focus of the reporting of the results. Two researchers (M.H. and H.H.I.) read and analysed the responses independently, before discussing and reaching a consensus on their content. Most of the questions in the employee questionnaire focused on the categories from the patient questionnaire or categories that corresponded to one of them. Accordingly, the categories in the patient questionnaire were used to structure the responses to the open-ended comments.

## 3 Results

There were 98, 101, 110, and 110 institutions that participated in the national patient surveys in 2013, 2014, 2015 and 2017, respectively. For each of the surveys the number of respondents were 978 (91%), 1,017 (91%), 1,184 (90%), and 1,173 (91%), respectively. As shown in Table 1, more than two-thirds of the patients were male, and their mean ages ranged from 36 to 38 years all four surveys. The mean age when they had developed substance dependence was around 20, and around 80% reported being single. Around half of the respondents had finished secondary school. Around 40% reported alcohol, medication, cannabis, and cocaine/amphetamine as the drug/substance they most frequently used, meaning that some reported several drugs/substances. The most commonly reported “length of stay at this institution” was 3–11 weeks, and more than 50% had either none or one previous admission prior to the current stay. For most of the background variables there were no statistically significant differences between the years. The very few statistically significant differences that were found (i.e., age, length of stay and medication as the most frequently used drug/substance used) were small. This gives a population that were similar over the four surveys.

**Table 1:** Descriptive statistics of patients and employees in the four surveys.

Patients	2013 (*n* = 978)	2014 (*n* = 1,017)	2015 (*n* = 1,184)	2017 (*n* = 1,173)	Change *p*^b^
Gender					
Male	628 (67.2)	681 (71.5)	795 (69.3)	797 (70.3)	0.225
Female	306 (32.8)	272 (28.5)	352 (30.7)	336 (29.7)	
Age, years	928 [36.5 (mean)]	928 [37.2 (mean)]	1,120 [37.5 (mean)]	1,109 [38.0 (mean)]	0.040
Age when substance dependence developed, years	919 [20.3 (mean)]	935 [20.6 (mean)]	1,118 [20.6 (mean)]	1,114 [20.6 (mean)]	0.890
Marital status					
Married/cohabitating	183 (19.7)	180 (19.1)	199 (17.4)	229 (20.3)	0.323
Single	744 (80.3)	763 (80.9)	945 (82.6)	898 (79.7)	
Education					
Primary school	383 (41.3)	367 (39.2)	435 (38.3)	432 (38.6)	0.876
Secondary school	434 (46.8)	452 (48.2)	561 (49.4)	550 (49.1)	
University or college	110 (11.9)	118 (12.6)	140 (12.3)	138 (12.3)	
Self-perceived physical health					
Excellent	60 (6.4)	67 (7.0)	71 (6.2)	65 (5.8)	0.653
Very good	192 (20.6)	189 (19.8)	248 (21.6)	238 (21.1)	
Good	330 (35.4)	333 (34.9)	418 (36.4)	425 (37.6)	
Quite good	225 (24.1)	247 (25.9)	290 (25.3)	281 (24.9)	
Poor	125 (13.4)	117 (12.3)	120 (10.5)	121 (10.7)	
Self-perceived mental health					
Excellent	47 (5.0)	49 (5.2)	52 (4.5)	55 (4.9)	0.965
Very good	145 (15.6)	158 (16.6)	197 (17.2)	205 (18.2)	
Good	317 (34.0)	308 (32.5)	367 (32.1)	345 (30.6)	
Quite good	260 (27.9)	276 (29.1)	344 (30.0)	340 (30.1)	
Poor	163 (17.5)	158 (16.6)	185 (16.2)	184 (16.3)	
Most frequently used drug/substance prior to this admission					
Alcohol	454 (46.4)	490 (48.2)	570 (48.1)	546 (46.5)	0.748
Medication	428 (43.8)	396 (38.9)	497 (42.0)	438 (37.3)	0.010
Cannabis	427 (43.7)	421 (41.4)	517 (43.7)	483 (41.2)	0.468
Cocaine/amphetamine	461 (47.1)	481 (47.3)	600 (50.7)	563 (48.0)	0.302
Heroin/morphine	256 (26.2)	266 (26.2)	321 (27.1)	300 (25.6)	0.865
Other	124 (12.7)	109 (10.7)	141 (11.9)	136 (11.6)	0.590
Length of stay at this institution					
0–2 weeks	144 (14.8)	138 (13.7)	164 (13.9)	134 (11.5)	0.001
3–11 weeks	377 (38.9)	387 (38.4)	470 (39.9)	448 (38.6)	
3–6 months	257 (26.5)	318 (31.5)	313 (26.6)	330 (28.4)	
7–12 months	147 (15.2)	131 (13.0)	176 (14.9)	162 (13.9)	
>12 months	45 (4.6)	34 (3.4)	55 (4.7)	88 (7.6)	
Previous admissions					
0	304 (32.5)	334 (35.2)	355 (31.1)	365 (32.3)	0.195
1	243 (26.0)	243 (25.6)	299 (26.2)	280 (24.8)	
2	167 (17.9)	147 (15.5)	174 (15.2)	197 (17.4)	
3–5	136 (14.6)	162 (17.1)	217 (19.0)	196 (17.3)	
>5	84 (9.0)	64 (6.7)	97 (8.5)	92 (8.1)	

Employees	2013 (*n* = 304)	2014 (*n* = 244)	2015 (*n* = 266)	2017 (*n* = 279)	Change *p*^b^
Gender					
Male	96 (32.7)	75 (31.8)	87 (34.5)	97 (34.9)	0.857
Female	198 (67.3)	161 (68.2)	165 (65.5)	181 (65.1)	
Age, years	298 [46.2 (mean)]	233 [46.8 (mean)]	246 [46.9 (mean)]	266 [47.7 (mean)]	0.362
Duration of working at the institution, years	300 [9.2 (mean)]	233 [9.2 (mean)]	246 [9.5 (mean)]	269 [10.0 (mean)]	0.628
Type of position					
Clinical work	163 (54.7)	113 (47.9)	119 (47.6)	138 (49.5)	0.301
Administration	156 (52.3)	115 (48.7)	123 (49.2)	139 (49.8)	0.833
Quality improvement/professional development	131 (44.0)	85 (36.0)	98 (39.2)	118 (42.3)	0.263
Other	38 (12.8)	37 (15.7)	41 (16.4)	37 (13.3)	0.557
Employee status					
Not manager	122 (40.9)	97 (41.3)	105 (41.8)	99 (36.1)	0.552
Middle manager	110 (36.9)	92 (39.1)	103 (41.0)	120 (43.8)	
Top manager	66 (22.1)	46 (19.6)	43 (17.1)	55 (20.1)	
Professional background					
Medical doctor	8 (2.7)	3 (1.3)	4 (1.6)	6 (2.2)	0.656
Psychologist	41 (13.9)	31 (13.2)	32 (12.9)	34 (12.2)	0.948
Nurse	79 (26.7)	71 (30.2)	57 (22.9)	75 (26.9)	0.343
Social educator	21 (7.1)	14 (6.0)	12 (4.8)	19 (6.8)	0.702
Child-care worker	32 (10.8)	21 (8.9)	26 (10.4)	29 (10.4)	0.906
Social worker	59 (19.9)	50 (21.3)	57 (22.9)	47 (16.8)	0.354
Other	97 (32.8)	67 (28.5)	85 (34.1)	95 (34.1)	0.508
Previously responded to this employee survey^a^					
Yes	NA	113 (48.3)	133 (53.0)	130 (46.9)	0.697
No	NA	52 (22.2)	53 (21.1)	66 (23.8)	
Do not know or remember	NA	69 (29.5)	65 (25.9)	81 (29.2)	

Data are *n* (%) values.

^a^ Question not asked in 2013.

^b^ Chi-square tests for categorical variables, one-way ANOVA for continuous variables. NA, not asked.

The numbers of employees who were recruited and who responded were 384 and 304, respectively, in 2013, 403 and 244 in 2014, 432 and 266 in 2015, and 416 and 279 in 2017 (response rates of 79%, 61%, 62% and 67% in the four years). Many of the employees participated in several of the surveys. In all four surveys, two-thirds of the respondents were female, and their mean age was around 47 years. The mean duration of work experience at the institution was 9–10 years (Table 1). More than 20% of the employees were nurses, social workers or had other professional backgrounds. Around 20% were top managers, while 40% were middle managers and 40% were not managers. Approximately half of the respondents cited clinical work and/or administration as their position type, while around 40% reported that they were involved in quality improvement or professional development.

Table 2 lists the results from all four survey years for the patient experience indicators and their underlying items. The scores across all years were best for the milieu indicator, at 75–77, followed by the outcome indicator (68–70) and the treatment-and-personnel indicator (60–63). The scores increased over time, with Bonferroni post-hoc correction showing that the differences between the years were mostly due to the scores in 2017 being higher than those in 2013 or 2014.

**Table 2:** Scores for patient experience indicators and their underlying items across years.

Treatment and personnel	2013 (*n* = 978)	2014 (*n* = 1,017)	2015 (*n* = 1,184)	2017 (*n* = 1,173)	Change *p*^a^
	60.4 ± 18.93	60.4 ± 18.32	61.8 ± 17.61	63.4 ± 17.19	<0.001
6: Have you had enough time for talking and contact with clinicians/personnel?	3.51 ± 1.06	3.48 ± 1.02	3.61 ± 0.97	3.67 ± 0.93	<0.001
7: Do you perceive that the clinicians/personnel have understood your situation?	3.65 ± 0.97	3.61 ± 0.98	3.66 ± 0.94	3.78 ± 0.91	<0.001
8: Have you had confidence in the professional competence of the clinicians/personnel?	3.68 ± 1.00	3.69 ± 0.93	3.72 ± 0.96	3.84 ± 0.94	0.001
9: Has one of the clinicians/personnel had primary responsibility for you?	3.75 ± 1.08	3.77 ± 1.07	3.86 ± 1.03	3.94 ± 0.99	<0.001
14: Has the information you have received regarding the treatment been satisfactory?	3.44 ± 1.00	3.42 ± 0.93	3.46 ± 0.91	3.51 ± 0.91	0.164
15: Have you had influence on your treatment?	3.53 ± 1.00	3.52 ± 0.95	3.62 ± 0.94	3.64 ± 0.94	0.006
16: Do you perceive that the treatment has been adjusted to your needs?	3.46 ± 1.02	3.44 ± 1.00	3.50 ± 0.98	3.57 ± 0.94	0.014
19: Have you received help for physical ailments or illness?	3.20 ± 1.11	3.14 ± 1.12	3.28 ± 1.10	3.25 ± 1.13	0.056
21: Has your access to psychologists been satisfactory?	3.13 ± 1.29	3.17 ± 1.29	3.22 ± 1.27	3.27 ± 1.22	0.125
22: Has your access to medical doctors been satisfactory?	3.33 ± 1.12	3.29 ± 1.11	3.33 ± 1.11	3.30 ± 1.11	0.826
31: Do you perceive that the clinicians/personnel have prepared you for the time after discharge?	2.97 ± 1.13	3.01 ± 1.10	3.00 ± 1.08	3.19 ± 1.11	<0.001
34: Do you perceive that the clinicians/personnel have helped you so you can achieve a meaningful life after discharge?	3.12 ± 1.17	3.17 ± 1.13	3.15 ± 1.11	3.29 ± 1.07	0.006
**Milieu**	**75.2 **± **16.61**	**74.8 **± **16.02**	**75.6 **± **15.29**	**77.1 **± **15.40**	**0**.**004**
4: Were you welcomed in a satisfactory manner when admitted to the institution?	3.98 ± 0.91	4.02 ± 0.87	4.08 ± 0.80	4.13 ± 0.81	<0.001
10: To what extent have you been met with courtesy and respect?	4.16 ± 0.83	4.09 ± 0.83	4.19 ± 0.79	4.26 ± 0.76	<0.001
25: Have you felt safe at the institution?	4.17 ± 0.81	4.15 ± 0.81	4.14 ± 0.80	4.17 ± 0.80	0.692
26: Has the institution arranged for contact with other patients in a satisfactory manner?	3.78 ± 0.96	3.81 ± 0.94	3.81 ± 0.95	3.86 ± 0.96	0.258
29: Have the meals at the institution been satisfactory?	3.92 ± 1.11	3.90 ± 1.06	3.92 ± 1.01	3.99 ± 1.01	0.166
**Outcome**	**67.9 **± **21.67**	**67.6 **± **20.70**	**68.8 **± **20.09**	**69.8 **± **20.26**	**0**.**074**
13: All in all, what benefit have you gained from the treatment at the institution?	3.85 ± 0.98	3.82 ± 0.94	3.87 ± 0.91	3.94 ± 0.92	0.032
35: All in all, is the help and treatment you receive at the institution satisfactory?	3.76 ± 0.95	3.79 ± 0.91	3.82 ± 0.90	3.87 ± 0.90	0.026
36: Do the help and treatment you receive at the institution improve your ability to understand your dependency problem?	3.64 ± 1.06	3.63 ± 1.04	3.72 ± 1.01	3.76 ± 1.00	0.007
37: Do the help and treatment you receive at the institution improve your ability to cope with your dependency problem?	3.61 ± 1.02	3.58 ± 0.98	3.64 ± 0.97	3.66 ± 0.97	0.265
38: Do the help and treatment you receive at the institution give you faith that your life will improve after discharge?	3.74 ± 1.02	3.72 ± 0.96	3.73 ± 0.96	3.73 ± 0.96	0.959

Data are mean ± SD values ranging from 0 to 100 (patient experience indicators) or 1 to 5 (underlying items).

^a^ One-way ANOVA with Bonferroni post-hoc correction.

Exploratory factor analysis confirmed that the five items measuring attitudes towards patient-experience surveys could be meaningfully reported as a unidimensional scale, for which Cronbach's alpha was 0.8 for all four years. As shown in Table 3, the five items constituting the attitude scale were all rated positively, with scores higher than 80 for all survey years, but a slightly lower score in the 2017 survey. Even though the one-way ANOVA showed a statistically significant change over time, the Bonferroni post-hoc correction revealed no significant changes in the attitude scale. However, when testing the single scale items, the scores on item 5 were significantly lower in 2017 than in the two earlier surveys. The results were somewhat less positive for whether the national patient-experience survey was useful for their institution, but they remained over 3. Forty percent of the respondents reported that their institution had implemented at least one improvement initiative after the patient survey of the previous year. This proportion increased to around 50% for 2015 and 2017. Most of the initiatives targeted the preparations for the post-discharge time, the treatment, the milieu and activities, and the therapists or the personnel.

**Table 3:** Selected employee-dependent variables, including the attitude scale and its underlying items, and areas targeted with initiatives after completing the patient-experience survey as reported by employees.

Attitude	2013 (*n* = 304)	2014 (*n* = 244)	2015 (*n* = 266)	2017 (*n* = 279)	Change *p*^b^
	83.1 ± 13.01	84.6 ± 11.93	84.0 ± 12.57	81.7 ± 12.17	0.047
1: Do you think it is important to involve patients in decisions regarding treatment and caring?	4.58 ± 0.57	4.60 ± 0.54	4.58 ± 0.56	4.55 ± 0.60	0.792
2: Do you think it is important to involve patients in health-care quality assessments?	4.40 ± 0.64	4.50 ± 0.60	4.50 ± 0.64	4.49 ± 0.59	0.160
3: All in all, do you think patient-experience surveys are important?	4.50 ± 0.64	4.54 ± 0.58	4.53 ± 0.60	4.41 ± 0.60	0.051
4: All in all, do you think patient-experience surveys can contribute to improvements in the health service?	4.28 ± 0.73	4.31 ± 0.69	4.26 ± 0.72	4.20 ± 0.71	0.315
5: Do you think patient-experience surveys can contribute to improving the medical quality of the health service?	3.85 ± 0.86	3.97 ± 0.77	3.93 ± 0.79	3.69 ± 0.73	<0.001
18: All in all, do you find that the patient-experience survey has been useful for your institution/department?^a^	*NA*	3.09 ± 0.98	3.34 ± 0.92	3.26 ± 0.93	0.012
17: Do you have knowledge of the report from the patient-experience survey with results from your institution/department?^a^					
Yes	NA	148 (62.7)	181 (72.1)	199 (71.6)	0.122
No	NA	64 (27.1)	55 (21.9)	57 (20.5)	
Not sure	NA	24 (10.2)	15 (6.0)	22 (7.9)	
11: Has your institution/department implemented initiatives to improve weaknesses identified by the patient-experience survey?^a^					
Yes, one	NA	22 (9.1)	24 (9.2)	25 (9.0)	0.027
Yes, multiple	NA	75 (31.0)	104 (39.8)	117 (41.9)	
No	NA	66 (27.3)	46 (17.6)	44 (15.8)	
Not sure	NA	79 (32.6)	87 (33.3)	93 (33.3)	
**Areas targeted with initiatives** ^a^	**2013**	**2014 (*n* = 92)**	**2015 (*n* = 122)**	**2017 (*n* = 142)**	
Reception and waiting time	NA	24 (26.1)	43 (35.2)	41 (28.9)	NA
Therapists/personnel	NA	39 (42.4)	50 (41.0)	62 (43.7)	NA
Treatment	NA	46 (50.0)	70 (57.4)	79 (55.6)	NA
Milieu and activity provision	NA	44 (47.8)	51 (41.8)	64 (45.1)	NA
Preparations for the time after discharge	NA	47 (51.1)	70 (57.4)	85 (59.9)	NA
Help from the municipality	NA	20 (21.7)	29 (23.8)	28 (19.7)	NA
Other	NA	9 (9.8)	10 (8.2)	14 (9.9)	NA

Data are mean ± SD values ranging from 0 to 100 (attitude scale) or 1 to 5 (underlying items), or *n* (%) values.

Each respondent could report multiple initiatives, and so the percentages do not sum to 100.

^a^ Question not asked in 2013.

^b^ Chi-square tests for categorical variables, one-way ANOVA for continuous variables.

One of the open-ended questions asked the employees to describe changes in their behaviour due to the results from the patient-experience surveys. The number of responses to this question increased over time, at 48 (12%), 74 (28%) and 94 (34%) in 2014, 2015 and 2017, respectively. Content analysis showed that 50 comments (23%) mentioned how employees had increased the user involvement in their work. Some gave examples of their institution developing a more formalized or structured approach to ensuring dialogue between patients and employees and leaders (e.g., “There has been a focus on making more time for contact persons to spend with their patients”). Others described that they now strived to include patients in more meetings or decisions (e.g., “Increased understanding and even more user management. We have, among other things, conducted group work, personnel and patients, where we have worked together on quality initiatives. We have more meetings with patients and fewer without”). Forty employees responded that they had tried to improve the areas identified from the patient-experience survey as being worse than expected or desired, or had targeted areas they knew they could improve (e.g., “In areas we scored lower than last year, we have changed the treatment in line with what the users want”). Others addressed the treatment, communication and information, the relationship between health personnel and patients, preparations for the post-discharge time, the milieu and activity provision, and providing more resources and positions and organization in general.

The employees who responded that they had implemented improvement initiatives after the previous patient survey were prompted to provide more detail. This open-ended question was answered by 33%–41% of the respondents each year. Many of the initiatives focused on organization of or methods employed at the institution. Two popular topics were routines, with 81 descriptions of different initiatives (27%) for ensuring that tasks were done in a timely manner and in a better/more systematic order than previously, such as regular meetings with interdisciplinary teams and patient assessments (e.g., “Procedure bindings are made, so that temps are also updated at all times”). The other popular topic was changing routines for the goals of patients or their treatment plans (e.g., “Therapists/patients work more systematically with a treatment plan and evaluations during the treatment”).

Many of these comments described how the treatments were structured and their contents. Routines for admitting new patients were highlighted in 58 comments (19%), such as introduction packages, contact with patients before they were admitted and buddy systems (e.g., “We have employed a collaboration consultant in the department. The task is to contact the patient and initiate measures before admission, inform about the treatment, initiate responsibility group meetings, etc”). Sixty comments (20%) described initiatives targeted at ensuring that patients were better prepared for the post-discharge time. These initiatives focused on the earlier onset of these preparations, and better routines for care after discharge (e.g., “Greater focus on preparation for the time after discharge is done with the patient and the support system—we have created a 100% position for a social worker and hired a psychologist. All patients should have an interdisciplinary team around them which consists of a therapist, a social worker, a nurse and a supervisor. The team should have 2 or 3 meetings with the patient during treatment in the clinic”). Other topics covered in these open-ended comments include a greater focus on activities for the patients, education and counselling for employees, patient involvement, working with friends or family, coordinating care, hiring more clinicians/personnel, and providing better information.

About half of the employees responded to the open-ended question regarding barriers to using the results from each patient-experience survey, with 40%–50% of them implying that they experienced no barriers. The most-common nuanced explanation of barriers was a lack of resources (*n* = 50, 13%), such as money, time, personnel and workload (e.g., “A lot of new hires mean that we have to carry out new training constantly. This is not good for continuity and relationships with patients”). Other employees (*n* = 26, 7%) found the patient-experience survey difficult to use due to the time between measurement and reporting, or had difficulty interpreting the results. These difficulties were due to the questions not fitting the patient group, or patients no longer being in the institution and new patients not agreeing with the results or knowing what the previous patients meant when responding (e.g., “It is difficult when a long time passes between the time of the survey and when the results come. Our patients partially distanced themselves from the critical findings we presented because they were not receiving treatment from us when the study had taken place”). Twenty-one employees (5%) replied that there were few respondents for their institution, making the limited results more uncertain. Other barriers included (i) a lot of other things going on at the institution, such as changes or reorganization, (ii) lack of interest or priorities from the leaders, (iii) other initiatives or surveys already being implemented, and (iv) the results being primarily positive.

## 4 Discussion

This study found that employees at residential institutions providing interdisciplinary treatment for substance dependence actively use the results from national patient-experience surveys in quality-improvement. The employees generally had positive attitudes towards patient-experience surveys, but were somewhat less positive about the usefulness of the national surveys for their own institutions. The most-common quality-improvement initiatives cited targeted areas that had the worst findings in the patient-experience survey, such as preparations for the post-discharge time, or to broader aspects of the treatment such as increasing patient involvement and developing the structure and content of the treatment. The experiences of the patients improved slightly over time, with the best experiences being reported in the most-recent survey for all indicators.

These findings are consistent with previous ones reported for paediatric departments in Norway (13), and also elsewhere where staff members considered patient-experience surveys as important for improvement efforts (22) User-experience surveys were considered important, with around half of the respondents reporting that they had implemented improvement actions, and addressed problems identified in the national survey. However, it is important to consider that the potential for improvement likely varied across institutions. Some institutions may have started with relatively high patient-experience scores in 2013, leaving less room for substantial improvement. In such cases, institutions may have only a few targeted areas where changes were necessary, and these areas may have been more challenging to address. On the other hand, institutions with lower initial scores may have had a broader range of areas for improvement, allowing for more targeted and actionable quality initiatives. This difference in the potential for improvement could explain why some institutions experienced more noticeable improvements in patient-experience scores over time, while others saw more modest changes.The employees reported several types of quality initiatives implemented at their institutions. A recent systematic review identified that many quality initiatives focused on organizational change and staff education (18). This is consistent with the current study finding a focus on more-systematic patient involvement in their day-to-day activities, and changing routines for both employees and patients. Several of the respondents mentioned education and counselling for employees, which constituted the second-most-common type of quality initiatives, a finding supported in the literature (18, 23). The most-common target areas reported by employees showed clear improvements in patient experiences, such as 50%–60% of employees reporting initiatives related to preparation for the post-discharge time, and both of the related patient-experience items improved significantly during the study period. This suggests causality, even though we should stress that the actual initiatives might have varied between institutions, and the modest nature of the changes at the national level.

The qualitative data showed that many employees focused their quality-improvement initiatives on areas with the worst results in the patient surveys. Additionally, employees often focused on areas where they knew they could improve their results, with more than half of the respondents reporting initiatives targeting “the treatment”. This is a similar approach as found by other studies (7, 12). This approach aligns with the potential for improvement perspective, as employees would naturally prioritize areas with the greatest potential for measurable impact. Nevertheless, institutions that started with fewer areas needing improvement may have faced greater difficulty in achieving significant changes. Still this high rate of reported initiatives supports the value of national surveys where the results can be reported at several levels of the health-care system. Each participating institution received the detailed survey results for their patients, allowing them to assess their relative scores and implement initiatives that are directly relevant to their patients and their own resources.

Several of the employees cited specific barriers to the utilization of data in quality initiatives. Such barriers have often been categorized into data-related, professional, and organizational ones (18, 24). In our study, the data-related barriers mentioned included the time between measurement and reporting, making the results less valid and/or difficult to utilize, as previously reported across different healthcare contexts (7). A small sample in an institution was also reported as a barrier by the employees that made the responses less trustworthy. Organizational barriers found in this study related to a lack of interest or priorities from the leaders or the institution already having many other things going on (either quality initiatives or their own surveys). Changes in the organization also reportedly increased the difficulty of prioritizing quality initiatives based on the national patient surveys. Some research has shown that employees may mistrust survey data (24, 25), but clinical scepticism was not prominent in the present study. The most commonly cited barrier reported by employees was a lack of resources to fully utilize the data. Although the employees were willing to engage with the survey findings, they reported to lack the time, personnel and financial resources to implement changes, a challenge echoed in international research (23, 26).

In January 2020 the NIPH started national continuous electronic measurements of the experiences of patient receiving interdisciplinary treatment for substance dependence and mental health care. All patients at the relevant institutions are asked to complete the patient-experience questionnaire a few days before being discharged. This method of data collection could overcome some of the barriers reported for this population, since the respondents will be at the same place in their clinical pathway (i.e., close to discharge) and the patient sample for each institution will be larger than in previous cross-sectional surveys, hence resulting in even more-timely reporting and more-robust statistical results. This data-collection method will continue until at least 2025, and there will be several opportunities to follow up the employee data and to analyse how the patient-experience scores change over time. Such research can reveal how and why patient experiences change over time, and which interventions are more effective in this population.

Previous systematic reviews of patient-experience surveys have summarized the reported barriers and facilitators (7, 12), which are consistent with the present finding of employees having positive attitudes towards patient-experience surveys. However, attitudes do not always lead to changes, and facilitators such as management support and a supportive culture are important for quality improvements, as well as ensuring that employees have sufficient time to review the results and plan the improvements to be implemented (7, 12, 18).

The four national patient-experience surveys and their preceding employee surveys were conducted consecutively between 2013 and 2017, and so the attitudes of employees might have been affected by collecting and reporting fatigue for externally initiated data. This may also have affected the response rates, as these decreased somewhat from the first survey to the remaining. Nevertheless, the employees generally reported very positive attitudes towards patient-experience surveys, and considered their associated workload to be relatively low. While the attitude score showed a negative trend over the four surveys, the scale score remained high and the changes over time were small.

### 4.1 Implications

Locally adapted and implemented quality-improvement initiatives have a potential for improving patient involvement and patient experience. As shown in this study, employees at the institutions gave several examples on how they tried to improve the patients’ experiences. Many of these local initiatives were focusing on becoming more patient-centred, involving patients earlier, and integrating patients’ views into both treatment and discharge planning. However, certain barriers—including limited time, financial constraints, and workforce shortages—hinder the full utilization of patient-experience data for quality improvement. The NIPH is actively addressing data-related barriers by improving the timeliness and accessibility of patient-experience data through continuous electronic measurement. However, institutional support is crucial for fostering a culture that embraces patient feedback as an integral part of quality improvement, and a lack of resources to use patient-experience data in local quality work is a barrier that needs to be addressed by the health services. All in all, the positive changes in patient experiences over time implies a fruitful collaboration between a national measurement initiative and local health care units. A final implication is thus the importance of accommodating OECDs principle of sustainability in national patient experience systems to monitor trends longitudinally (27) and to secure continuous feedback to the quality improvement work at the local health care units.

### 4.2 Strengths and limitations

The NIPH has been responsible for the four national patient-experience surveys and the four employee surveys. While this has provided an opportunity to follow the populations over time, individual patients were discharged from the institutions after different periods, sometimes earlier than planned, which made it difficult to track the patients in these surveys. The employees could theoretically have been followed across the years, but the national surveys were treated as stand-alone surveys rather than as yearly follow-ups. This was due to the nature of the surveys and how they were commissioned, and the permissions necessary to link individual data from multiple years were not applied for.

We originally planned to analyse the data at institution level, including to determine whether interventions described in one year induced changes in patient experiences in the subsequent surveys. Many of the institutions were small and we recruited only selected employees, resulting in the number of responses at the institution level being too small to conduct analysis at that level. Many employees reported implementing quality initiatives to improve patient experiences, but only small changes were seen in the national quality indicators. Given the large number of participating institutions and a range of initiatives implemented, it will be difficult to register changes in patient experiences on the national level. Furthermore, a systematic review found that studies focused on smaller numbers of initiatives were more likely to measure effects (18). Many of the employees in the present study reported implementing several initiatives, which may have confounded their effectiveness. While it was not possible to analyse data at the institution level in this study, it is likely that larger changes in patient experiences would be found when analysing larger samples. Nevertheless, positive changes in the quality indicators were found from the first to the last patient survey.

Overall, employees expressed positive attitudes towards patient-experience surveys, though this sentiment may have been influenced by the specific roles of the respondents. The recruitment strategy primarily targeted employees directly involved in quality assurance and improvement initiatives, which could naturally result in more favorable views towards the surveys, given the direct relevance to their work responsibilities. Employees not engaged in these initiatives might have different attitudes, which were less represented in the study. The recruitment criteria specifically asked project managers to recruit the following employees: department managers, institution managers, quality advisors, and one or two employees responsible for quality assurance and improvement. “Type of position” and “professional background” were both questions where the employees could tick more than one answer, giving an accumulated percentage higher than 100. This focused selection aimed to gather insights from employees directly involved in decision-making and quality initiatives at their institutions. The approach ensured a distribution among both leadership and frontline staff as well as a balanced representation of non-managerial staff, ensuring that the employee survey captured perspectives from across the organizational hierarchy. Thus, the survey responses reflected perspectives from key employees engaged in quality improvement across different levels of the organizational hierarchy, type of position and professional background.

Even though the employee surveys revealed mainly positive attitudes towards patient-experiences surveys and that many had implemented quality initiatives, it is difficult to know how much they influenced the patient experiences. It is a known limitation that cross-sectional surveys cannot measure cause-and-effect relationships. The variations across the participating institutions and other factors besides patient-experience surveys such as other initiatives, legislation and plans may affect how institutions treat their patients. Randomized controlled trials are the gold standard for revealing which interventions cause the greatest changes in patient experiences. The methodology and aims of our surveys meant that we did not have control over other variables that could influence both the employees and the experiences of the patients.

The long time periods between the patient-experience surveys, the reporting of results and the following employee surveys may have resulted in some employees experiencing difficulties recalling what had been done at their institution. Nevertheless, this was a known risk, and since there is always a time delay before an intervention produces any effect, the NIPH considered that the employee survey should be applied some time after reporting the results to allow for the implementation and local recording of possible interventions and their early effects.

## 5 Conclusions

This study found positive changes in patient experiences with interdisciplinary treatment for substance dependence across the yearly national cross-sectional surveys. Also, the employees had positive perceptions of the patient-experiences surveys. Even though their usefulness scores were lower, the results were still positive, with about half of respondents indicating that the institutions had implemented initiatives based on the results. The yearly surveys have followed patient and employee populations for which systematic knowledge and studies are still scarce, and hence constitute a unique source of data for on-going measurements. The design of this study makes it impossible to infer whether the reported initiatives induced the changes in patient experiences, but the most-common target areas reported by employees showed clear improvements in patient experiences.

This study presents unique data in several respects: all patients from all institutions in Norway were invited to participate in the patient surveys, and several employees from the same institutions were invited to share their attitudes towards such surveys, as well as to report on if and how they used the data in local quality work.

Future studies should perform follow-up assessments so that patient experiences can be measured before and after implementing local improvement initiatives in order to determine which interventions are the most effective. The impact of this research could be increased by conducting institution-specific follow-up surveys.

## Data Availability

The raw data supporting the conclusions of this article will be made available by the authors, without undue reservation.
